# A structure-based protocol for learning the family-specific mechanisms of membrane-binding domains

**DOI:** 10.1093/bioinformatics/bts409

**Published:** 2012-09-03

**Authors:** Morten Källberg, Nitin Bhardwaj, Robert Langlois, Hui Lu

**Affiliations:** ^1^Department of Bioengineering, University of Illinois at Chicago, Chicago, IL 60607, USA; ^2^Program in Computational Biology and Bioinformatics, Yale University, New Haven, CT 06520, USA; ^3^Howard Hughes Medical Institute, Columbia University Medical Center, 10032, New York, NY, USA; ^4^Shanghai Institute of Medical Genetics, Children Hospital of Shanghai, Shanghai, China; ^5^Key Lab of Embryo Molecular Biology, Ministry of Health, China; ^6^Shanghai Lab of Embryo and Reproduction Engineering, Shanghai, China

## Abstract

**Motivation:** Peripheral membrane-targeting domain (MTD) families, such as C1-, C2- and PH domains, play a key role in signal transduction and membrane trafficking by dynamically translocating their parent proteins to specific plasma membranes when changes in lipid composition occur. It is, however, difficult to determine the subset of domains within families displaying this property, as sequence motifs signifying the membrane binding properties are not well defined. For this reason, procedures based on sequence similarity alone are often insufficient in computational identification of MTDs within families (yielding less than 65% accuracy even with a sequence identity of 70%).

**Results:** We present a machine learning protocol for determining membrane-targeting properties achieving 85–90% accuracy in separating binding and non-binding domains within families. Our model is based on features from both sequence and structure, thereby incorporation statistics obtained from the entire domain family and domain-specific physical quantities such as surface electrostatics. In addition, by using the enriched rules in alternating decision tree classifiers, we are able to determine the meaning of the assigned function labels in terms of biological mechanisms.

**Conclusions:** The high accuracy of the learned models and good agreement between the rules discovered using the ADtree classifier and mechanisms reported in the literature reflect the value of machine learning protocols in both prediction and biological knowledge discovery. Our protocol can thus potentially be used as a general function annotation and knowledge mining tool for other protein domains.

**Availability:** metador.bioengr.uic.edu

**Contact:** huilu@uic.edu

## 1 INTRODUCTION

Signal transduction networks formed by specific protein–protein and protein–lipid interactions are a primary means by which the cell transmits information from its external environment to intracellular recipients. One vehicle driving the intracellular signal transduction speed beyond that of simple diffusion is the selective and reversible binding of so-called peripheral proteins to membrane surfaces within the cell (Hurley, 2006; Pawson and Scott, 1997). By redistributing cytosolic proteins to membranes in response to the onset of signaling events a *de facto* compartmentalization of the cellular space takes place, allowing for greater proximity among communicating parties, thereby facilitating interaction (Hurley and Misra, 2000). The importance of this mode of signal transduction is underlined by the fact that more than 10% of human protein kinases contain at least one lipid-binding module (Hurley, 2006). The ability to identify and understand peripheral proteins and the physical factors causing their co-localization at membranes is thus pivotal in uncovering the dynamics governing signaling regimens.

Peripheral proteins are most commonly scaffold proteins containing one or more domains that associate with lipid-head groups, thereby anchoring the entire protein structure near the lipid surface (Cho and Stahelin, 2005; DiNitto *et al.*, 2003; Pawson and Scott, 1997). An increasing number of ubiquitous and structurally distinct domains have been found to display lipid binding properties, collectively referred to as membrane-targeting domains (MTDs). MTDs have been identified in the following families: C1 (Cho, 2001; Sanchez-Bautista *et al.*, 2006; Yang and Kazanietz, 2003), C2 (Cho, 2001; Nalefski and Falke, 1996; Rizo and Sudhof, 1998), PH (Ferguson *et al.*, 2000; Lemmon and Ferguson, 2000), FYVE (Fab1/YOTB/Vac1/EEA1) (Stenmark *et al.*, 2002), PX (phox) (Xu *et al.*, 2001), ENTH (Epsin N-terminal homology)(Camilli *et al.*, 2002), and recently PDZ domains (Chen *et al.*, 2012). Despite their highly similar intra-family folds, not all domains in these families possess membrane-targeting properties. In fact, a diverse array of overlapping intra-family functions exists, spanning from protein–protein interaction to structural support and potentially enzymatic activity (Hurley, 2006).

Numerous experimental techniques have been used to identify novel MTDs (Cho, 2001; DiNitto *et al.*, 2003) revealing details on binding mechanisms and orientation (Ball *et al.*, 1999; Corbalan-Garcia *et al.*, 2003). Genome-scale identification and characterization of MTDs does, however, remain labor intensive and expensive. To this end *in silico* protocols offer a high-throughput complement to wet-laboratory methods, allowing for rapid characterization of thousands of domains. Membrane-binding properties are inherently difficult to predict, as they are often not determined by well-defined sequence motifs or a specific structural composition. PDZ domains were, for instance, found to have highly diverging membrane-binding behavior despite high sequence similarity (Chen *et al.*, 2012) and PH domains span a large range of binding affinities though being structurally very similar (Lemmon and Ferguson, 2000; Singh and Murray, 2003).

In previous works from our laboratory, machine learning protocols for distinguishing MTDs from a general body of cytosolic protein domains know to have no membrane-binding activity were constructed using support vector machines (SVM) (Bhardwaj *et al.*, 2006) and later on extended using other classifiers (Langlois *et al.*, 2007). By representing each domain as a numerical vector of feature values derived from structural data, a classification model achieving 90% accuracy in separating binding and non-binding domains was constructed. There are, however, two issues of this model to be addressed. First, while performing well when separating MTDs from cytosolic protein domains of unrelated fold, the model does not provide similar performance in separating binding and non-binding domains within any specific family. As we will demonstrate, intrafamily classification is in fact a very hard problem as even highly similar domains display different membrane binding properties. Second, the constructed SVM model does, to a great extent, function as a ‘black-box’ classifier giving little insight as to how the different calculated features play together in producing the final classification of a domain's binding properties.

In this work, we construct a series classification models for separating membrane-binding domains from domains with other activity within families. Our focus is on C1, C2 and PH domain families, as domains from these three families have been found to be key players in a number of signaling pathways. We are, however, not merely interested in constructing models for classification, as such models are of limited utility in explaining the predicted behavior in a manner that leads to experimentally testable hypothesis. Rather, we want to provide both a confident assessment of a given binding behavior and a body of biological evidence supporting the classification label. The goal is to go from data mining to knowledge mining revealing the specific mechanisms responsible for observable higher level behavior. To this end, we take advantage of a new ensemble-based classifier, namely the alternating decision tree (ADtree) algorithm (Freund and Mason, 1999). The ADtree relates to other classification tree algorithms such as CART and C4.5 (Quinlan, 1986) by quantifying the relationship between features as a combination of rules each representing a binary decision on a feature. The ADtree is based on the boosting technique but is at the same time a tree structure representable as a conjunction of rules all contributing a real-valued additive evidence toward classification. The final classification decision is thus determined by a committee voting scheme based on the real values evidence presented by each rule traversed in the tree by a given domain. This scheme makes representation of the classifier as a spare and easily interpretable tree structure possible, a feat recently demonstrated in studies for identifying DNA-binding proteins and characterizing their binding mechanisms (Langlois *et al.*, 2007; Langlois and Lu, 2010).

The article is organized as follows: first, we give the intuition behind the features used to represent the individual domains in a form suitable for constructing machine learning protocols. We then construct classification models based on SVM and ADtree to separate intra-family binding and non-binding domains. Finally, we analyze the individual rules used in determining membrane targeting behavior in the three domain families, in terms of experimentally known binding mechanisms.

## 2 METHODS

### 2.1 Dataset

Special care was taken when selecting the positive and negative examples in the datasets used, as both the instance groups come from the same domain family (Bhardwaj *et al.*, 2007). After reducing the sequence identity to 70%, a total of 303 sequences were left. Each of these instances was then examined manually and classified as positive (binding) and negative (non-binding) based on their functions, sub-cellular location and similarity with other sequences. The final statistics for the three datasets used for training are given in Table 1. As a reference, the total number of annotated domains for each family in PFAM is also provided, to underline the ubiquitous nature of all three families. The 70% cutoff was chosen as sequence similarity at this level does not result in conservation of membrane-binding properties, as illustrated in the Results section.

**Table 1.** Dataset statistics for the three domain families

Domain	Binding	Non-binding	MaxSimilarity	PFAM
C1	33	22	70%	1536
C2	63	27	70%	4666
PH	70	88	70%	4125

Only a subset of the domains in the constructed datasets have an experimentally solved structure available. For the rest of the cases (74% of all domains), we construct homology models using the RaptorX server (Källberg *et al.*, 2012). For all modeling cases, we have found at least one template with more than 30% sequence identity, thus ensuring the quality of the modeled structure.

### 2.2 Classifiers and evaluation

Models were constructed using two binary classification procedures, namely the ADtree (Freund and Mason, 1999) and SVM (Cortes and Vapnik, 1995). Both are supervised classifiers, for which a model is trained on a labeled training dataset (training mode) and thereafter applied to predict new examples without further parameter tuning (prediction mode). Casting the problem in a binary classification framework, we refer to each protein domain as an instance, with the *i*^th^ instance consisting of a feature vector *x_i_* ∈ [1 × *n*] and a label *y_i_* ∈ {0,1}, with *n* denoting the feature count. Both algorithms described construct a function, *g*(*x*), that minimizes the empirical risk of misclassifying an instance, under the assumption that all instances are drawn with respect to the same (unknown) probability distribution. In the following, we limit ourselves to describing conceptual details of the used algorithms, referring the reader to cited works for technical details.

The SVM methodology facilitates the derivation of a classification hyperplane (a hyperplane separating positive and negative cases) for nonlinear problems by working in a vector space of higher dimension than that of the original feature space. The separating hyperplane, *wx* + *b*, can be found by numerically solving the following quadratic optimization problem:

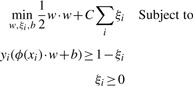

Where *C* and *ξ_i_* are cost parameters associated with misclassification, and *ϕ*(*x_i_*) is a non-linear mapping function. Rewriting the above problem in the dual form, the kernel-trick, specifying a function giving the inner-product of two vectors in a higher dimensional vector space, can then be applied and an optimal separating hyperplane can be found in this new vector space. In this work, we tested Gaussian, Sigmoid and polynomial kernel function and found that the Gaussian to give the best results, thus whenever SVM results are reported, it refers to SVM using a Gaussian kernel function.

The ADtree uses the boosting methodology (Freund and Mason, 1999) in the same manner as other successful classification schemes such as Adaboost C4.5 (Quinlan, 1986) but has the advantage of producing models that are easily representable as a tree with a limited number of nodes (often fewer than 20), without sacrificing predictive power. This is achieved by constructing a tree that is a conjunction of rules which all provide an additive evidence toward a given instance being classified as positive or negative, depending on the evaluations of the rules (true or false). In addition to providing the classification label, the tree score of an instance (the margin score) can be interpreted as a measure of confidence in the classification label. Unlike traditional tree models obtained from algorithms such as C4.5, the classification of instances by ADtree is thus not determined by a single path traversed in the tree but rather by a collection of paths. The tree is made up of two types of nodes prediction nodes, represented by ellipses, and splitter nodes, represented by rectangles. Each splitter node is associated with a real-valued number indicating the rule condition: if the feature represented by the node is less than or equal to the condition value for a given instance, the prediction path will go through the left child node, otherwise the path will go through the right child node. The final classification score produced by the tree is found by summing the values from all the prediction nodes reached by the instance, with the root node being the precondition of the classifier. If the summed score is greater than zero, the instance is classified as positive, otherwise, as negative.

We use our in-house machine learning workbench MALIBU for the construction and validation of models, giving a uniform interface for comparison and analysis of their performance (Langlois *et al.*, 2007).

We measure the performance of the constructed classification models using the following metrics: accuracy (Acc) defined as the ratio of true prediction to the total number of prediction, sensitivity (Sen) defined as the probability that a true example is classified as true and specificity (Spe) defined as the probability that a negative example is classified as negative. The classification result of an instance in a binary classification can be fall into four categories: true positive (TP), false positive (FP), true negative (TN) and false negative (FN). Using these counts, the three metrics are approximated by: Acc = (TP+TN)/(TP+TN+FP+FN), Sen = TP/(TP+FN) and Spe = TN/(TN+FP)

Further, we use the area under the receiving operator characteristic curve (AUC ROC). AUC ROC is defined as the area under the (1-specificity, sensitivity)-curve, with each point corresponding to a specific threshold for class separation; a value of 1 performs perfect over the entire range of threshold values, with a random classifier having an AUC value of 0.5.

To provide a benchmark for the expected performance we use *n*-fold cross validation (*n*-CV). In *n*-CV, one randomly divides the original dataset into *n* equally sized bins, each classifier is then trained *n* times using *n* – 1 subsets. The omitted subset in each round is used for estimating the evaluation metrics of interest, the average of which is thus based on evaluation over all instances. For this work, 20-CV was used.

### 2.3 Features

The association with membranes is known to be driven by a combination of general lipid binding mechanisms and the binding of key residues with specific lipid head groups. The general association mechanisms are modeled by quantifying the chemical and physical properties of the domain structure as a collection of ‘patches’ on the solvent exposed surface (SES). A patch is a well-defined area of a given property on the surface, i.e. an area of all positive electrostatic potential or a region of conserved hydrophobicity; here, we use the area of five largest patches in each category. Further, the surface propensities of the 20 amino acids are included as features for a total of 35 structural features. The steps of patch growing detailed below are outlined in (Källberg and Lu, 2009). The basic idea is as follows: First, the surface is defined as a collection of neighboring triangles; second, a numerical representation of the quantity of interest is associated with each triangle and finally the patches that are most highly correlated with the function of the structure are defined.

Assume that each triangle on the surface is associated with a numerical value corresponding to the quantity that forms the basis for patch growing. We will denote this value for a triangle *t* by *t.*val and the distance between the centroids of triangles *t*_1_ and *t*_2_ by dist(*t*_1_,*t*_2_). Furthermore, *t.*neigh will denote the neighbor triangles of *t*, meaning those that share an edge with *t*, and *t.*included will be a boolean flag indicating whether a given triangle has been included in a patch. The collection of patches is then found by repeating the following recursive procedure until all surface triangles have been included in a patch: choose a random triangle that has not yet been included in a patch and extend the patch by adding neighbor triangles that satisfy |*t*1.val – *t*2.val| *< C*, where *C* is a constant.

To do the patch growing, we need to assign values from the quantity of interest to each triangle on the SES. In this work, we use three quantities: (i) the electrostatic potential obtained from solving the Poisson-Boltzmann (PB) equation using APBS (Baker *et al.*, 2001). The spatial potential values are mapped onto the surface by taking a weighted average of the eight discrete data points closest to the point 1 A from the triangle surface in the direction of its normal vector. (ii) hydrophobicity values are assigned to the surface based on the Kyte–Doolittle value of the amino acid that gave rise to the triangle of interest (Kyte and Doolittle, 1982) and (iii) hydrogen-bonding is mapped to the surface by determining if an atom is capable of forming a hydrogen bond, indicated by setting *t.*val = 1.

In addition to the patch-growing procedure, features solely based on statistics from the full collection of domain sequences are used. A score for each domain sequence is obtained by its similarity to other sequences in the dataset, this is done using a recursive functional classification (RFC) matrix inspired by Park *et al.* (2008). A multiple sequence alignment of all domain sequences (both binding and non-binding) is created using a Hidden Markov Model profile (for the C1, C2 and PH domain, the PFAM models PF00130, PF00168 and PF00169 were used, respectively). On the basis of alignment, we calculate the probability of observing amino acid *a* at location *i* in the alignment. Denoting the probability for binding and non-binding cases by *P*_*a,i*,+_ and *P*_*a,i*,−_, respectively, each entry in the the RFC matrix is given by:

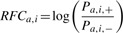


Thus a positive/negative entry in the matrix indicates that the presence of amino acid *a* at location *i* is evidence toward the domain being membrane binding/non-binding. We can summarize the evidence for a giving domain sequence *S* as being binding in the following score:

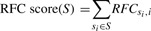


For the sequence features, we do, however, choose to decompose the RFC matrix into a series of residue subsequence features of length 3–6, to be able to more specifically pin point the exact local variation that was evidence for classification. Rather than using all possible rules, we include the 25 rules that provide the greatest degree of discriminatory power in the training set. The rule locations are selected through an initial bootstrap generation of several RFC matrices and a ranking of the rules that have the highest potential RFC score. The subsequence rules are thus intended to complement general membrane-binding mechanisms by identifying subsets of residues that correlate with specific-binding modes. In general, the quantifying local environment conservation has been shown to be of great utility in identifying remote similarity properties. Recently, procedures focusing on local environment have been used with great success in the identification of DNA-binding protein domains (Langlois and Lu, 2010) and in more general purpose protocols for remote homology detection (Biegert and Soding, 2009).

## 3 RESULTS

The contribution of this work is 2-fold. First, we show that machine learning models based on the sequence and structure features introduced below perform significantly better than procedures based on sequence homology in separating MTDs from non-MTDs within families. Second, we demonstrate how ADtree models not only perform comparable to a SVM-based models but also present us with specific evidence for the classification label allowing us to interpret the model within the context of current experimental observations.

**Fig. 1.**
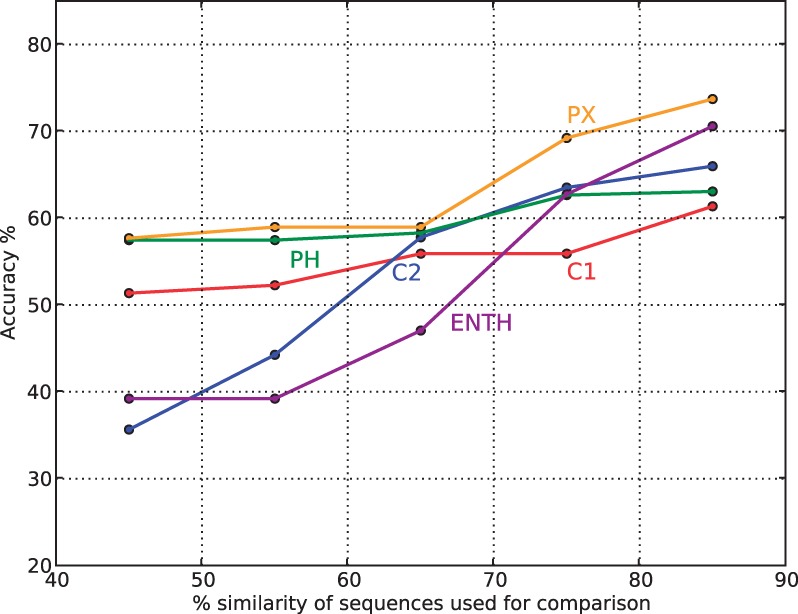
Performance of a sequence-based nearest-neighbor classification procedure. Accuracy measures for classification of membrane binding properties for five domain families using consensus of the three nearest neighbors for each domain. The accuracy for each family is depicted at varying levels of maximum sequence similarity allowed between instances in dataset for each domain family

To further illustrate the difficulty of the current classification task of intrafamily separation, a simple unsupervised classification scheme aimed at predicting the membrane-binding behavior of a domain based on the binding behavior of closely related homologs is fashioned. A sequence is predicted to be binding or non-binding from the majority vote decision of its three closest related sequence neighbor [as defined from a BLAST search (Altschul *et al.*, 1990)]. Figure 1 depicts the prediction accuracy for the procedure as a function of varying levels of the maximum sequence similarity allowed between domains in the dataset. It is evident that even at maximum sequence similarity levels as high as 85%, accuracies of no more than 75% can be achieved for any family, indicating the need for more sophisticated procedures to confidently identify MTDs within families.

### 3.1 Overall classifier performance

Table 2 compares the performance of SVM, ADtree and the sequence-based nearest neighbor protocol for three domain families. In all families, the two structure-based machine learning protocols perform significantly better domain separation than the sequence based nearest neighbor procedure at the 70% sequence identity level. For C1 domains both SVM and ADtree improve on the accuracy of the sequence-based method by more than 30% points, with both achieving accuracy rates in the 90% range. The SVM protocol does, however, have a 6% better AUC ROC than the ADtree due to a better balance between sensitivity and specificity, making it the strongest classifier for this family.

**Table 2..** Performance comparison of models for C1, C2 and PH domain families, bolded values indicate the best performing classifier, details on validation metrics are given in the Methods section

Family	Algorithm	Acc.	Sen.	Spe.	AUC ROC
C1	ADTree	0.891	**0.939**	0.818	0.887
	SVM	**0.907**	0.909	**0.909**	**0.957**
	Seq Sim	0.57	0.43	0.78	—
C2	ADTree	**0.856**	**0.889**	**0.778**	**0.879**
	SVM	0.792	0.838	0.7	0.878
	Seq Sim	0.63	0.61	0.7	—
PH	ADTree	0.861	0.824	0.853	0.905
	SVM	**0.867**	**0.843**	**0.886**	**0.939**
	Seq Sim	0.64	0.64	0.62	—

Inspecting the models constructed for C2 and PH domains, we again observe a far better performance of the machine learning methods over the sequence homology-based classifier, with accuracy improvements of 22% points for both families. Comparing the SVM and ADtree models for the C2 domain families, a similar performance over the entire specificity range is observed as the AUC ROC of the two models is almost identical, although the ADtree achieves a higher accuracy when comparing the points of best class separation for the two classifiers. For the PH domain family, we again observe the two classifiers performing comparable, with a small advantage to the SVM algorithm of 3% points as measured by AUC ROC. In sum, the results show that for the problem at hand the ADtree models perform comparable or only slightly worse than SVM, indicating almost no loss in performance when using a model that has the feat of human interpretability.

Though not presented here, we have experimented with machine learning protocols relying solely on sequence-derived features. Although these protocols did show better performance than the nearest neighbor homology-based procedure used as bench mark above, they never achieved accuracies higher than 75%, which is significantly less than what was achieved by using both sequence-and structure-based features.

### 3.2 Knowledge mining

**Fig. 2.**
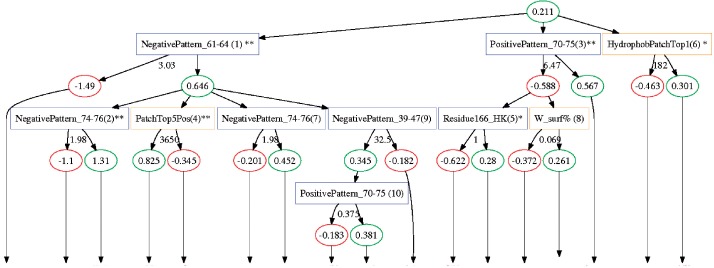
The ADtree model constructed for the C1 domain family. A single rule is represented by two elliptical prediction nodes and a rectangular splitter nodes. Each splitter node is associated with a real valued number indicating the rule condition, if the condition is true/false the path traversed by an instance will go through the left/right child node and accumulate the score in this node toward the overall classification of a domain. Splitter nodes colored in blue stem from sequence feature, whereas yellow ones stem from structure features

Here, we use the ADTree model to discover the rules that distinguish binding and non-binding domains. A graphical representation of the ADtree model constructed for the C1 domain family is depicted in Figure 2, yellow and blue splitter nodes signify structure and sequence-based feature rules, respectively. Sequence-based rules are divided into positive and negative patterns indicating subsequences in the domain family alignment for which there is a high/low RFC score for binding/non-binding sequences. Feature names are followed by a number in parenthesis indicating the order in which the rules are added to the model, a measure that can be interpreted as an importance ranking of the rules. The fact that top-ranked rules are a mix of sequence and structure-based features, indicates that both groups are adding orthogonal predictive power to the model, a feat also observed in the tree models for C2 and PH domains (not shown). In the following subsections, we will interpret key rules in the three classification models in terms of their biological meaning in driving reversible membrane binding; the importance of ranking is used when referring to specific rules in each model.

#### 3.2.1 C1 model

C1 domains are cystine-rich modules of approximately 50 amino acids in length, first discovered in protein kinase C (PKC) and subsequently found in signaling families such as chimaerins, RasGRPs and diacyglycerol kinases (Colon-Gonzalez and Kazanietz, 2006).

**Fig. 3.**
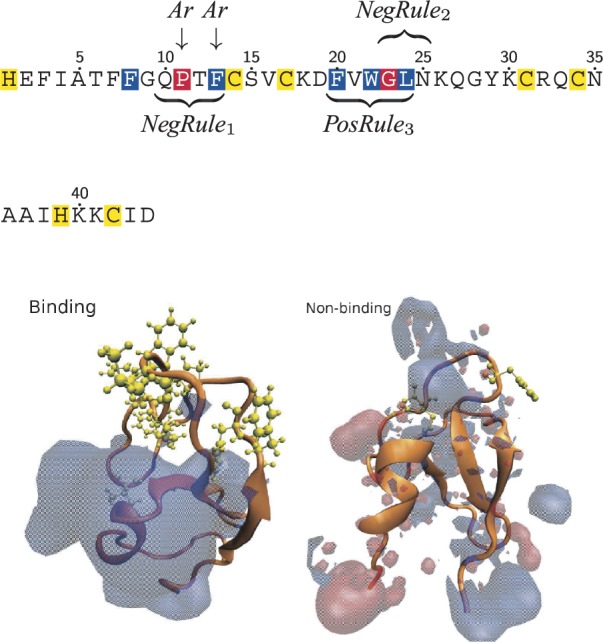
Rules learned for the C1 family. The sequence for PKC*δ*-C1a is used for illustrating key rules, the residue coloring used is membrane binding (blue), DAG binding (red) and Zinc-binding (yellow). Structure models for a binding case and non-binding case are shown, with the positive and negative electrostatic isosurfaces color in blue and red, respectively. In addition, hydrophobic residues are highlighted in yellow

The sequence of the known binding case PKC*δ*-C1a is used in Figure 3 for illustrating key rules learned for the entire C1-family. Membrane binding of C1 domains is known to be driven by specific binding of diacylglycerol (DAG) and phorbol esters in the membrane, as well as the association of key residue with the membrane surface and coordinated binding of Zn^2+^, these features are highlighted in the PKC sequence both. Rules 2 and 3 overlap with the second group of membrane and DAG-binding residues indicating that two different kinds of function conservation appear here: one associated with membrane binding and one associated with other activity. NegRule1 is observed to be high scoring if residues 11 and 13 are not aromatic, correlating well with the experimental evidence for binding, as interfacial penetration of the lipid bilayer has been found to be driven by aromatic residues (Langlois *et al.*, 2007; Stahelin *et al.*, 2003).

In addition to the conservation of specific sequence groups, two global structure mechanisms are also discovered by the C1 tree. Rules 4 and 6 indicate that if the cumulative size of the five largest electrostatic patches and the size of the largest hydrophobic patch are greater than specific threshold values, it is indicative of membrane binding. This observation correlates well with the fact that certain C1 domains are known to deeply penetrate the hydrophobic membrane core on binding, an interaction that is only energetically favorable if non-polar surface-residue exists. Similarly, a somewhat positively charged surface is necessary for the initial recruitment of the domain to anionic lipid surfaces (Hurley, 2006). The two mechanisms are illustrated in the lower part of Figure 3 by structure data from a binding and a non-binding C1 case, with electrostatic positive and negative isosurfaces superimposed and hydrophobic residues highlight in yellow. For the binding-case, there is a large well-defined bulk of positive electrostatics and a cluster of hydrophobic residues, whereas the non-binding only display sporadic regions of positive charge.

#### 3.2.2 C2 model

**Fig. 4.**
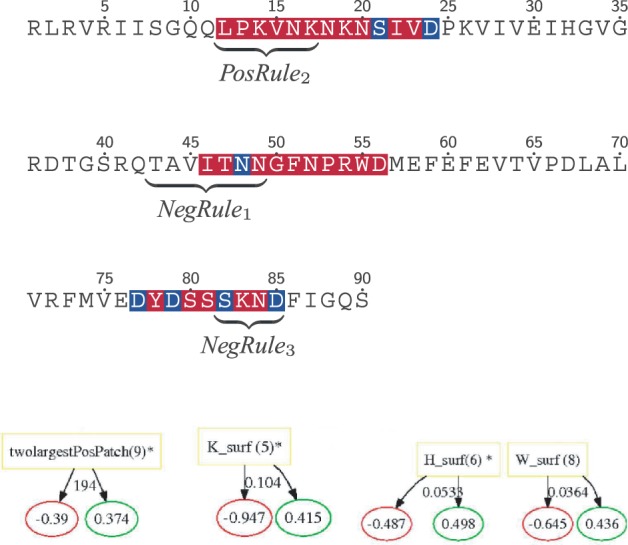
Rules learned for the C2 family. The sequence for PLC*δ*1 is used for illustrating key sequence rules, and the residue coloring used is Ca^2+^ binding side chain (blue) and Ca^2+^-binding region (red). Further, four structure rules from the C2 model are shown

Most C2 domains require activation by divalent Ca^2+^ ions to bind to membranes with high affinity and show low affinity toward lipids otherwise (Cho and Stahelin, 2005; Hurley, 2006). In the majority of cases, binding of Ca^2+^-ions dramatically enhances the positive electrostatic potentials around the Ca^2+^-binding region that mediates the association with the anionic lipids (termed as an electrostatic switch) (Chen *et al.*, 2012) or induces a conformational change that accelerates binding (Kulkarni *et al.*, 2002; Shao *et al.*, 1998). As illustrated in Figure 4, the discovered sequence rules from the C2 domain model all overlap with Ca^2+^-binding regions. Interestingly, the first negative rule also overlaps with regions suggested to be involved in protein-protein interactions in PKC*ε* (Brandman *et al.*, 2007) indicating the conservation of functional properties other than membrane binding in this region.

Further, a number of structure rules are used in the C2 model. Cationic residues on the surface (in corporation with Ca^2+^ bridging) are important for anionic-lipid selectivity (i.e. synaptotagmin) (Cho and Stahelin, 2005). We observe this in rules indicating a threshold on positive surface patches and surface propensity on K. Finally, selectivity for the lipid-head group PC in C2 domains is achieved through aromatic and aliphatic surface residues (i.e. observed in cPLA2), represented in the model as high surface propensities of amino acids H and W being indicative of membrane binding (Cho, 2001).

#### 3.2.3 PH model

**Fig. 5.**
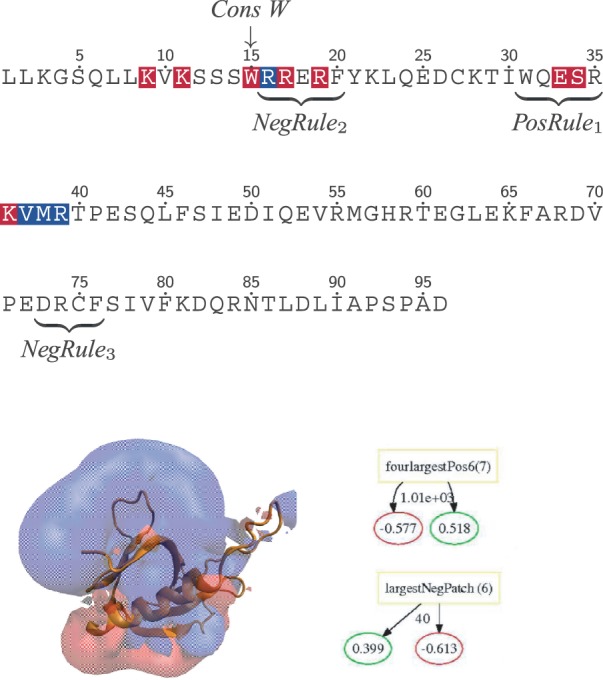
Rules learned for the PH family. The sequence for PLC*δ*1 is used for illustrating key rules, and the residue coloring used is membrane binding (blue) and PIP_3_/PIP_2_ binding (red). A structure model for a binding case is shown with the positive and negative electrostatic isosurfaces color in blue and red, respectively. In addition, two key structure rules are depicted

PH domains are recruited to membranes by phosphatidylinositol lipids such as phosphatidylinositol (3,4,5)-trisphosphate (PIP_3_) and phosphatidylinositol (4,5)-bisphosphate (PIP_2_) and are, in example, found in *βγ*-subunits of heterotrimeric G proteins (Wang *et al.*, 1994) and PKC (Yao *et al.*, 1994). For the PH domain family, binding often occurs in two steps, an initial association is driven by non-specific electrostatic interactions followed by specific binding to anionic lipids (Hurley and Misra, 2000). Key rules from the PH family model, illustrated in Figure 5, agree well with this overall process of membrane association. Two structure rules both presenting minimum cutoffs on the size of electrostatic patches are present. As observed in other families, a large positive patch is indicative of binding. Interestingly, a smaller negative patch is also positively associated with binding in the case of PH domains. This apparent inconsistency that both positive and negative charge promotes membrane binding can be explained from the local arrangement of the electrostatic potential depicted in Figure 5. Here, we see that the negative region is on the opposite side of the membrane associating surfaces, thus the repulsion of this side to a negatively charged membrane can help correctly position the domain relative to the membrane, a mechanism previously hypothesized (Hurley, 2006).

In addition to the electrostatic mechanism for correct spatial orientation, sequence rules 1 and 2 overlap with residue experimentally determined to be important in phosphatidylinositol and general membrane binding (Hurley, 2006). The third sequence rule mapped does not immediately correlate with any known residues important for binding but does contain two positively charged residues that may be important in binding, thus these residues may constitute a novel functional region.

## 4 DISCUSSION AND CONCLUSION

This work touches on two key challenges of computational biology: How do we efficiently organize and classify the vastly expanding body of data produced by experimentalists; and of even greater importance, how do we transform this data into biological knowledge in the form of testable hypotheses. It can be argued that simple rule mining would be an appropriate option to deduce classification rules. However, it is widely believed that discriminative approaches are far superior to generative ones given their simplicity. Moreover, discriminative classifiers have been shown to have a lower asymptotic error (Bishop, 2006). Further, ADtrees have the ability to elicit more uncorrelated rules (by definition) that cover diverse features of the data, although of course being limited to the feature space initially defined.

The graphical models built for the three families highlight the general rules and features that set binding instances of peripheral proteins apart from non-binding ones. Although some of these features are in agreement with previous studies, novel features are also proposed. In general, we find that structural features, such as a specific cutoff for the size of positive electrostatic surface patches, are found in models for all domains and thusly constitutes general mechanisms driving membrane binding. Sequence-based features on the other hand, are more important in expressing unique binding motifs for each family as they are rooted in local regions of the domains.

Characteristics elucidated from the rules learned in this work can be used to guide further experimental studies. For example, mutation of certain amino acids that are statistically over-represented in important rules could be suggested as pointers for experiments (such as aromatic residues for C1/C2 domains). Similarly, if feasible, features such as overall charge on the domain could be tinkered with by multiple mutations of charged residues. Such guided studies are expected to reduce the effort and time required to reveal the mechanisms and features used by peripheral proteins and highlights the value of knowledge mining over the ‘black-box’-type approaches that are often used in classification of biological data.

*Funding*: This work was in part supported by the Chicago Biomedical Consortium with support from The Searl Funds at the Chicago Community Trust. M.K. thanks FMC Technologies Fund Fellowship for support. H.L. acknowledges support from the National Natural Science Foundation of China (Grant No. 31071167).

*Conflict of Interest:* none declared.
